# Descriptive epidemiology of long‐term injuries in jump racing Thoroughbreds in Great Britain

**DOI:** 10.1111/evj.70129

**Published:** 2025-11-24

**Authors:** Sophia McDonald, Kristien L. P. Verheyen, Yu‐Mei Chang, Sarah E. Allen

**Affiliations:** ^1^ Department of Pathobiology and Population Sciences Royal Veterinary College Hatfield Hertfordshire UK; ^2^ Department of Comparative Biomedical Sciences Royal Veterinary College London UK

**Keywords:** fracture, horse, incidence rates, racehorse, tendon injury

## Abstract

**Background:**

Race‐related injuries in horse racing, especially those requiring extended recovery, are a welfare concern and threaten the sport's social licence. Previous studies predominantly report on fatalities; however, serious non‐fatal musculoskeletal injuries often end horses' racing careers or have a high recurrence risk. No recent studies have described or quantified long‐term injuries (LTIs) in racing Thoroughbreds, which is essential to inform targeted risk prevention strategies.

**Objectives:**

To describe the types, frequencies and incidences of LTIs in British jump racing.

**Study Design:**

Retrospective cohort.

**Methods:**

Analyses included all starts made in British hurdle and steeplechase races between 1 May 2018 and 30 April 2024. An LTI was defined as any non‐fatal musculoskeletal injury incurred during racing, following which the horse had a minimum 90‐day break from racing. LTIs were described by clinical diagnosis(es), body region(s) and anatomical structure(s) affected. Incidence per 1000 starts was calculated overall, by race type and year. Stratified incidence rates were calculated for selected injury types by age, sex, season, going and faller status.

**Results:**

There were 918 LTIs recorded in 898 horses. The overall incidence of LTIs was 5.5 per 1000 jump starts (95% CI 5.2–5.9), with 5.2 per 1000 hurdle starts (95% CI 4.8–5.7) and 6.0 per 1000 steeplechase starts (95% CI 5.4–6.7). Tendon and ligament injuries (TLIs) were the most common, accounting for 72.9% of LTIs in hurdle starts and 70.6% in steeplechase starts. Firmer going, summer and falling in a race were associated with a higher LTI incidence.

**Main Limitations:**

Classification of LTIs commonly relied upon presumptive diagnoses.

**Conclusions:**

The incidence of LTIs was higher in steeplechase than hurdle starts, and TLIs are an important cause of morbidity in British jump racehorses. This study provides a benchmark for ongoing LTI surveillance in jump racing, against which to monitor the effects of future interventions.

## 1 INTRODUCTION

Racehorses participating in jump races have been identified to be at an elevated risk of sustaining a musculoskeletal injury (MSI) on the racecourse compared to racehorses participating in flat races.^1^, ^2^, ^3^, ^4^, ^5^ Race‐related injuries are a welfare concern and threaten the sport's social licence to operate, as public unease regarding the use of animals in sport and concern for the welfare of racehorses increases.^6^, ^7^, ^8^, ^9^, ^10^ Minimising risk requires an understanding of different injury types so that targeted risk factor analyses can be undertaken and temporal trends can be monitored, allowing for the development of preventative measures.^11^, ^12^

In Great Britain (GB), 35.2% (3518/9988) of races in 2024 were held over jumps.^13^ Jump racing in GB occurs year‐round, with the ‘core’ jump season featuring the major jump racing festivals running from October to April each year. There are three main types of jump (National Hunt) races in GB: steeplechasing, where horses compete over larger fences; hurdling, where horses race over smaller, collapsible hurdles; and National Hunt flat (NHF) racing, designed for horses bred for jump racing to gain racecourse experience before they race over obstacles.

Even though MSI is reported as the leading cause of death in Thoroughbred racehorses,^4^, ^14^, ^15^, ^16^, ^17^, ^18^, ^19^ not all MSIs are fatal. These injuries span a broad range of severities, from minor strains and sprains that have little impact on a horse's training or racing schedule to severe tendon and ligament injuries or fractures requiring extended recovery and rehabilitation periods.^20^ The latter are those that often result in premature termination of a horse's racing career^10^, ^14^ due to a high risk of re‐injury^21^, ^22^, ^23^ or because retirement is necessary if the prognosis for returning to racing is poor.^14^, ^24^

A number of studies have described the incidence of tendon and ligament injuries (TLIs) in jump racehorses in GB,^2^, ^6^, ^25^ consistently finding that the superficial digital flexor tendon (SDFT) was the most commonly affected structure, which has consequently been studied separately.^26^, ^27^, ^28^ The most recent published studies of SDFT injuries in British jump racing, using data from 2000 to 2009, estimated the incidence of SDFT injuries at 6.1 per 1000 hurdle starts and 6.3 per 1000 steeplechase starts.^6^ Non‐catastrophic fractures are less clearly defined in the literature and are often included in studies using broad case definitions.^2^, ^15^, ^29^ The development of techniques to repair fractures under standing sedation and local anaesthetic has increased treatment options for numerous fracture types,^30^ therefore potentially increasing the number of non‐fatal fracture cases. The most recent estimates of overall fracture incidence in jump racehorses in GB are over a decade old, and were reported as 3.2 per 1000 starts in hurdle races and 5.0 per 1000 starts in steeplechase races.^6^

No recent studies have estimated the incidence of LTIs in British jump racing. Therefore, the objectives of this work were to provide up‐to‐date incidence estimates of race‐related LTIs and to describe and quantify the types and frequencies of LTIs in hurdle and steeplechase racing in GB. This is essential to direct risk factor analyses for these injuries that will inform risk prevention strategies and for measuring the effectiveness of resulting interventions.

## 2 MATERIALS AND METHODS

### 2.1 Study design and data collection

A retrospective cohort study of all steeplechase and hurdle race starts made by Thoroughbred racehorses on British racecourses between 1 May 2018 and 30 April 2024 was conducted. Details of all LTIs, recorded by official veterinary officers in the British Horseracing Authority's (BHA's) purpose‐built injury database, were extracted. These data were combined with routinely recorded start data, including horse demographics and performance history, race conditions and course information, collated by Weatherbys' Racing Services (www.weatherbys.co.uk).

### 2.2 Definition of long‐term injuries

A long‐term injury (LTI) was defined as any non‐fatal musculoskeletal injury incurred during racing, following which the horse had a minimum 90‐day break from racing. All injuries were diagnosed by the attending racecourse veterinary surgeons at the racecourse, with diagnosis primarily based on clinical examination alone. Racecourse veterinary surgeons marked injuries as LTIs at the time of injury on race day, but injuries were recoded as short‐term injuries if the horse returned to racing within 90 days.

Long‐term injuries were categorised as either bone injuries, joint injuries, TLI, gait observation or other. The bone injury category included fractures and possible fractures. Joint injuries included dislocation and effusion, but excluded fractures into the joint. The category ‘gait observations’ included lame horses or those with poor/abnormal action, but where no further diagnosis was made. The ‘other’ category included injuries affecting the skin and muscles, such as wounds, lacerations, unlocalised inflammation or soreness and hoof wall avulsions.

Horses could sustain more than one type of LTI in the same start, if diagnoses corresponded to several of the different categories as listed above. Where this occurred and for the calculation of incidence rates stratified by injury type, each injury type was considered separately, although for overall LTI incidence these were considered as one injury for that horse start.

### 2.3 Data analysis

In this study, the unit of observation was a race start; that is, a horse that crossed the starting tape, and one horse could make multiple starts during the study period. Horses that were withdrawn prior to starting in the race were excluded from calculations. The total number of starts per horse was summarised as the median and interquartile range (IQR).

Long‐term injuries were described as counts and percentages and stratified by injury and race type. Bone injuries and TLIs were described by their location on the body (distal limb, proximal limb, or other body areas) and specific anatomical site(s).

Incidence of LTI was calculated as the number of injuries per 1000 starts for all jump starts and then reported separately for hurdle and steeplechase starts in all further analyses. The incidence of LTI was calculated for the overall study period and separately for six 12‐month periods from May to April (referred to as racing year). Incidence rates were also calculated across the entire study period for each injury type. For all LTIs, bone injuries and TLIs, incidence rates were stratified by age (in years: 3–5, 6–8, 9–11, ≥12), sex (male, female), season (spring [March, April, May], summer [June, July, August], autumn [September, October, November], winter [January, February, December]), going (heavy, soft, good to soft, good, good to firm, firm) and faller status (whether or not the horse fell in the race). Associated 95% confidence intervals (CIs) for each incidence rate were calculated using the exact (Clopper‐Pearson) binomial method.

All analyses were conducted in Stata version 18 (StataCorp LLC).

## 3 RESULTS

During the six‐year study period, there were 166,190 jump starts made by 21,336 horses over 3101 days and on 41 racecourses. These horses were trained by 1236 trainers and ridden by 857 jockeys. In the hurdle dataset, 18,947 horses made 106,423 starts in 11,358 hurdle races, with a median of 4 (IQR 2–7) starts per horse during the study period. In the steeplechase dataset, 9527 horses made 59,767 starts in 8188 steeplechase races, with a median of 4 (IQR 2–9) starts per horse during the study period.

### 3.1 Long‐term injuries

The overall incidence of LTIs was 5.5 per 1000 jump starts (95% CI 5.2–5.9). There were 558 (60.8%) LTIs in hurdle races and 360 (39.2%) in steeplechase races. The incidence of LTIs was 5.2 per 1000 starts (95% CI 4.8–5.7) in hurdle races and 6.0 per 1000 starts (95% CI 5.4–6.7) in steeplechase races.

**FIGURE 1:**
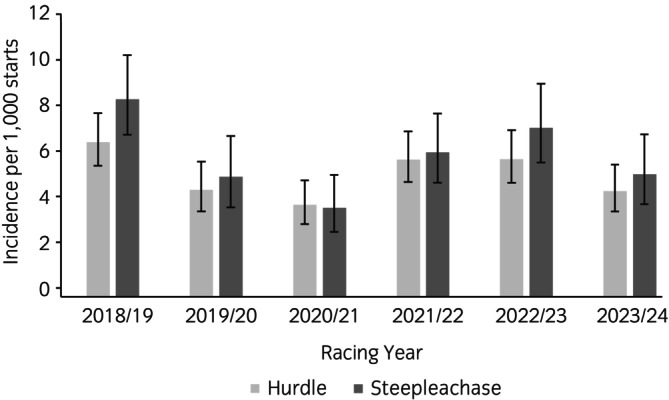
Incidence of long‐term injuries experienced by jump (steeplechase and hurdle) racing Thoroughbreds in Great Britain as reported by race day veterinary surgeons between 1 May 2018 and 30 April 2024, by racing year (May to April). Error bars represent 95% confidence intervals.

Figure 1 summarises the incidence rates of LTIs by racing year for hurdle and steeplechase starts over the study period.

Table 1 summarises the number and incidence of race‐related LTIs recorded over the study period. There was a total of 918 recorded LTIs, involving 898 horses, over 778 individual races and across 455 race days. Twenty horses had multiple occasions of race‐related LTI during the study period. Of these, twelve horses sustained two TLIs, two horses sustained two bone injuries, three horses sustained a bone injury and a TLI, and three horses sustained a bone injury and other MSI. There were 14 LTIs where multiple diagnoses were made, seven each in hurdle and steeplechase starts. Five of these involved injury to both bone and tendon or ligament, four involved two bone injuries, two involved a TLI and other MSI, one involved a bone injury and other MSI, one involved a bone injury and joint injury, and one involved two TLIs.

**TABLE 1:** Number and incidence of long‐term injuries experienced by jump racing Thoroughbreds in Great Britain as reported by race day veterinarians between 1 May 2018 and 30 April 2024.

Injury type/condition	Total, *n* (% of total)^a^	Hurdle starts		Steeplechase starts	
		Number (%)^a^	Incidence per 1000 starts (95% CI)	Number (%)^a^	Incidence per 1000 starts (95% CI)
Total long‐term injuries	918	558	5.2 (4.8–5.7)	360	6.0 (5.4–6.7)
Tendon and ligament injuries	661 (72.0)	407 (72.9)	3.8 (3.5–4.2)	254 (70.6)	4.2 (3.7–4.8)
Bone injuries	207 (22.5)	123 (22.0)	1.2 (1.0–1.4)	84 (23.3)	1.4 (1.1–1.7)
Joint injuries	9 (1.0)	4 (0.7)	0.04 (0.01–0.1)	5 (1.4)	0.09 (0.03–0.2)
Other musculoskeletal injuries^b^	38 (4.1)	21 (3.8)	0.2 (0.1–0.3)	17 (4.7)	0.3 (0.2–0.5)
Gait observations^c^	12 (1.3)	8 (1.4)	0.08 (0.03–0.1)	4 (1.1)	0.07 (0.02–0.2)

^a^ Totals will equal >100% as more than one diagnosis could be reported per LTI.

^b^ Other musculoskeletal injuries included wounds and lacerations, inflammation and soreness and hoof wall avulsions.

^c^ Gait observations included lame horses, but where no further diagnosis was made.

### 3.2 Tendon and ligament injuries

Tendon and ligament injuries were the most common type of LTI observed on race day (72.9% of all LTIs in hurdle starts and 70.6% in steeplechase starts). There were 661 TLIs, 407 (61.6%) in hurdle starts and 254 (38.4%) in steeplechase starts. The incidence of TLI was 3.8 per 1000 starts in hurdle races and 4.2 per 1000 starts in steeplechase races (Table 1).

Most TLIs were to the SDFT in both hurdle (*n* = 343, 92.7% of distal limb TLIs) and steeplechase starts (*n* = 196, 89.9% of distal limb TLIs). The incidence of SDFT injuries was 3.2 (95% CI 2.9–3.6) per 1000 starts in hurdle races and 3.3 (95% CI 2.8–3.8) per 1000 starts in steeplechase races. Injuries to the suspensory ligament (*n* = 23, 6.2% in hurdle starts; *n* = 15, 6.9% in steeplechase starts) were the next most common (Table 2). In 63 TLIs, the affected structure was not listed. In one hurdle start, both the superficial and deep digital flexor tendons were affected. In seven TLIs, another LTI from a different category was also diagnosed in the same start. There were 14 cases of bilateral forelimb TLI and one case of bilateral hindlimb TLI.

**TABLE 2:** Description of bone and tendon and ligament long‐term injuries associated with jump (hurdle and steeplechase) racing in Great Britain between 1 May 2018 and 30 April 2024, by anatomical region and injury location.

	Region	Injury location	Total number (%)	Number in hurdle starts (%^a^)	Number in steeplechase starts (%^a^)
Bone injury	Distal limb		106^b^	65^b^	42
		P1	6 (5.6)	4 (6)	2 (5)
		P3	2 (1.9)	0	2 (5)
		MC3/MT3 Condylar	25 (23.4)	19 (29.)	6 (14)
		MC3/MT3	5 (4.7)	3 (5)	2 (5)
		MC2/4 or MT2/4	4 (3.7)	1 (2)	3 (7)
		Sesamoid	8 (7.5)	5 (8)	3 (7)
		ACB	39 (36.4)	21 (32)	18 (43)
		Carpal or Tarsal	15 (14.0)	11 (17)	4 (10)
		Radius or Ulna	3 (2.8)	1 (2)	2 (5)
	Proximal limb		74	42	32
		Scapula	7 (10)	2 (5)	5 (16)
		Tibia or Fibula	4 (5)	3 (7)	5 (16)
		Humerus	4 (5)	3 (7)	1 (3)
		Pelvis	55 (74)	30 (71)	25 (78)
		*Tuber coxae*	4 (5)	4 (10)	0
	Other		30	17	13
		Skull	7 (23)	5	2
		Costal	6 (20)	2	4
		Vertebrae	13 (43)	9	4
		Unspecified	4 (13)	1	3
Tendon and ligament injury	Distal limb		588^c^	370^c^	218
		SDFT	539 (91.7)	343 (92.7)	196 (89.9)
		DDFT	4 (0.7)	1 (0.3)	3 (1.4)
		Suspensory ligament	38 (6.4)	23 (6.2)	15 (6.9)
		Seasmoidian ligaments	3 (0.5)	2 (0.5)	1 (0.5)
		ALDDFT	4 (0.7)	1 (0.3)	3 (1.4)
	Proximal limb		11	3	8
		Gastrocnemius tendon	2	0	2
		*Peroneus tertius*	3	0	3
		*T. Achilis/*SDFT	6	3	3
	Other	Unspecified	63	35	28

Abbreviations: ACB, accessory carpal bone; ALDDFT, accessory ligament of the deep digital flexor tendon; DDFT, deep digital flexor tendon; MC2/4, second or fourth metacarpal bone; MC3, third metacarpal bone; MT2/4, second or fourth metatarsal bone; MT3, third metatarsal bone; P1, proximal phalanx; P3, distal phalanx; SDFT, superficial digital flexor tendon.

^a^ Within each anatomical region; percentages can be >100% as horses could have more than one injury within the same category.

^b^ One distal limb bone injury included more than one bone.

^c^ One distal limb tendon and ligament injury included more than one tendon/ligament.

Overall, 287 (43.4%) TLIs were reported as severe (breakdowns or ruptures), with a slightly higher percentage in hurdle starts (*n* = 186, 45.6%) compared to steeplechase starts (*n* = 101, 39.8%). Moderate strains made up 38.9% (*n* = 257) of TLIs overall, accounting for 41.3% (*n* = 105) of TLIs in steeplechase starts, and 37.3% (*n* = 152) in hurdle starts. Slight strains made up only 2.6% of TLIs (*n* = 17). In 51 (7.7%) cases the tendon or ligament was lacerated or severed; in 33 (5.0%) cases the tendon or ligament was avulsed or dislocated. The injury severity/condition was not listed for 17 TLIs.

### 3.3 Bone injuries

There was a total of 207 bone injuries, 123 (59.4%) in hurdle starts and 84 (40.6%) in steeplechase starts. These accounted for 22.0% and 23.3% of all LTIs in hurdle and steeplechase starts, respectively. The incidence of non‐fatal bone injuries was 1.2 per 1000 starts in hurdle races and 1.4 per 1000 starts in steeplechase races (Table 1). Bone injuries were described as fractures (58.8%), possible fractures (40.8%) or unspecified (0.4%). In one hurdle start and three steeplechase starts, more than one bone was affected: pelvis and accessory carpal bone (ACB); proximal and medial sesamoid; scapula and costal; scapula and skull. In seven bone injuries, another LTI from a different category was also diagnosed in the same start. Distal and proximal limb injuries accounted for 106 (50.5%) and 74 (35.3%) bone injuries, respectively (Table 2); one distal limb injury included more than one bone.

In the distal limb, the ACB was the most commonly affected structure in both steeplechase (*n* = 18, 42.9%) and hurdle (*n* = 21, 32.3%) starts. The incidence of ACB fractures was 0.2 (95% CI 0.1–0.3) per 1000 hurdle starts and 0.3 (95% CI 0.2–0.5) per 1000 steeplechase starts. This was followed by third metacarpal or metatarsal (MC3/MT3) condylar fractures, though these accounted for a higher percentage of distal limb bone injuries in hurdle starts (*n* = 19, 29.2%) in comparison to steeplechase starts (*n* = 6, 14.2%). The incidence of MC3/MT3 condylar fractures was 0.2 (95% CI 0.1–0.3) per 1000 hurdle starts and 0.1 (95% CI 0.04–0.2) per 1000 steeplechase starts. Most proximal limb fractures were of the pelvis in both hurdle (*n* = 30, 71.4%) and steeplechase (*n* = 25, 78.1%) starts. The incidence of pelvic fractures was 0.3 (95% CI 0.2–0.4) per 1000 hurdle starts and 0.4 (95% CI 0.3–0.6) per 1000 steeplechase starts.

### 3.4 Other long‐term injuries

There were nine joint injuries, four in hurdle starts and five in steeplechase starts (Table 1). The incidence of joint injuries was <0.1 per 1000 starts in both hurdle and steeplechase races (Table 1). Most joint injuries were enlargements and effusions (*n* = 5), followed by sprains (n = 3), avulsions or dislocations (*n* = 1). The fetlock was the most commonly affected joint (*n* = 6). Other structures affected were the hock joint (n = 1) and intercarpal joint (*n* = 2).

The incidence of other MSIs was 0.2 per 1000 starts in hurdle races and 0.3 per 1000 starts in steeplechase races (Table 1). The majority of other MSIs were lacerations or wounds (*n* = 30, 79%), six (16%) were inflammation or soreness, and two (5%) were hoof wall avulsions. The fetlock was the most commonly affected structure (37%, *n* = 14), followed by the tendon area (26%, *n* = 10). Twelve lacerations or wounds (32%) had possible or confirmed involvement of the digital flexor tendon sheath. In four (11%) cases, the structure was not described further than the affected limb.

Gait observations were recorded in 12 LTIs. The incidence of gait observations was <0.1 per 1000 starts in both hurdle and steeplechase races (Table 1).

### 3.5 Stratified incidence rates

**FIGURE 2:**
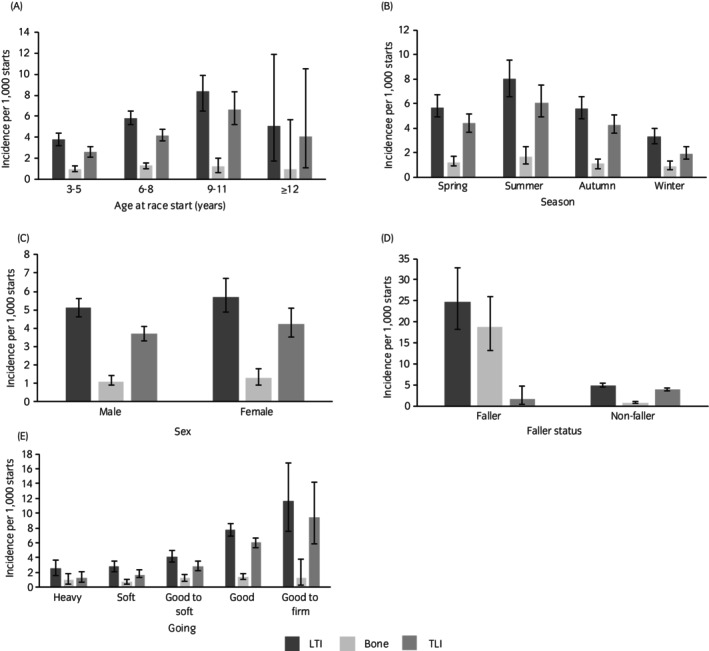
Incidence of long‐term injury (LTI), bone injury and tendon and ligament injury (TLI) events experienced by hurdle racing Thoroughbreds in Great Britain as reported by race‐day veterinary surgeons between 1 May 2018 and 30 April 2024, stratified by (A) age at race start; (B) season; (C) sex; (D) faller status; and (E) going. Error bars represent associated 95% confidence intervals for each incidence rate.

**FIGURE 3:**
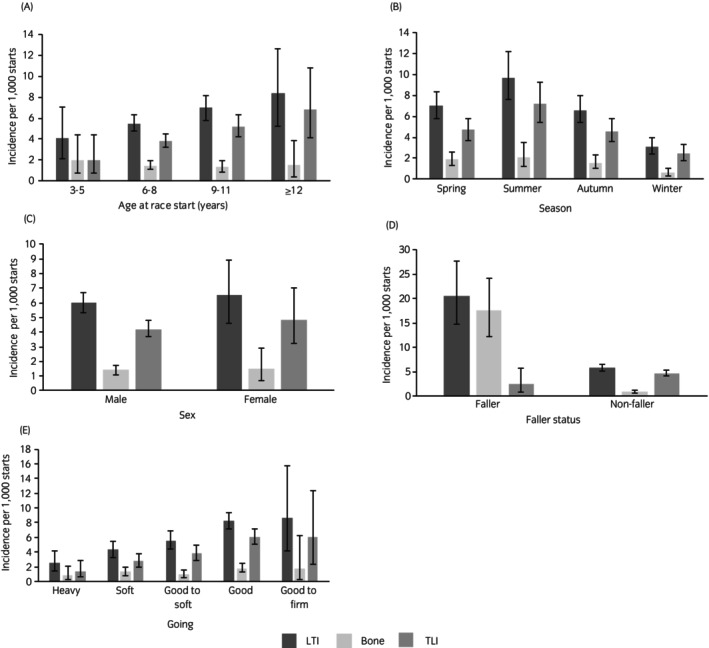
Incidence of long‐term injury (LTI), bone injury and tendon and ligament injury (TLI) events experienced by steeplechase racing Thoroughbreds in Great Britain as reported by race‐day veterinary surgeons between 1 May 2018 and 30 April 2024, stratified by (A) age at race start; (B) season; (C) sex; (D) faller status; and (E) going. Error bars represent associated 95% confidence intervals for each incidence rate.

Stratified incidences of LTI, bone injury and TLI for overall jump starts are reported in Table 3. Figures 2 and 3 summarise the incidences of LTI, bone injury and TLI for hurdle and steeplechase starts respectively, stratified by age, sex, season, going and faller status (exact incidence rates, associated confidence intervals and number of starts are reported in Table S1).

**TABLE 3:** Incidence of long‐term injury (LTI), bone injury and tendon and ligament injury (TLI) events experienced by jump (steeplechase and hurdle) racing Thoroughbreds in Great Britain as reported by race‐day veterinary surgeons between 1 May 2018 and 30 April 2024, stratified by sex, age, going, season and faller status.

Variable category	Starts, *n* (%)	Incidence per 1000 starts (95% CI)		
		LTI	Bone injury	TLI
Age (years)				
3–5	44,298 (26.7)	3.8 (3.3–4.4)	1.0 (0.7–1.4)	2.6 (2.1–3.1)
6–8	88,082 (53.0)	5.7 (5.2–6.2)	1.3 (1.1–1.6)	4.0 (3.6–4.5)
9–11	30,207 (18.2)	7.3 (6.4–8.4)	1.2 (0.8–1.6)	5.7 (4.8–6.6)
≥12	3603 (2.2)	7.5 (4.9–10.9)	1.4 (0.5–3.2)	5.6 (3.4–8.6)
Sex				
Male	133,842 (80.5)	5.4 (5.1–5.8)	1.2 (1.0–1.4)	3.9 (3.6–4.2)
Female	32,348 (19.5)	5.8 (5.1–6.8)	1.3 (1.0–1.8)	4.3 (3.6–5.1)
Season				
Spring	46,727 (28.1)	6.2 (5.5–7.0)	1.4 (1.1–1.8)	4.5 (3.9–5.1)
Summer	21,729 (13.1)	8.6 (7.4–9.9)	1.8 (1.3–2.5)	6.5 (5.5–7.6)
Autumn	45,135 (27.2)	6.0 (5.3–6.7)	1.2 (0.9–1.5)	4.4 (3.8–5.1)
Winter	52,599 (31.6)	3.2 (2.8–3.8)	0.8 (0.6–1.1)	2.1 (1.7–2.5)
Going				
Heavy	14,771 (8.9)	2.5 (1.8–3.5)	0.8 (0.5–1.5)	1.3 (0.7–2.0)
Soft	40,730 (24.5)	3.3 (2.8–3.9)	0.9 (0.6–1.3)	2.1 (1.7–2.6)
Good to soft	40,771 (24.5)	4.6 (4.0–5.3)	1.1 (0.8–1.4)	3.1 (2.5–3.7)
Good	66,398 (40.0)	7.9 (7.2–8.6)	1.5 (1.3–1.9)	6.0 (5.4–6.6)
Good to firm	3491 (2.1)	10.6 (7.5–14.6)	0.6 (0.5–3.3)	8.3 (5.6–11.9)
Firm	29 (<0.1)	0 (0–119.4)	0 (0–119.4)	0 (0–119.4)
Horse fell in race				
Yes	3861 (2.3)	22.5 (18.1–27.7)	17.9 (13.9–22.6)	2.1 (0.9–4.1)
No	162,329 (97.7)	5.5 (5.2–5.9)	0.8 (0.7–1.0)	4.0 (3.7–4.3)

*Note*: Associated 95% confidence intervals (CIs) were calculated for each incidence rate.

There was a general increase in the point estimate for the incidence of LTIs and TLIs with increasing age in steeplechase starts, but in hurdle starts the incidence was lower in horses ≥12 years old. Females had a higher incidence of LTIs, bone injuries and TLIs than males in both hurdle and steeplechase starts. The incidence of LTIs, bone injuries and TLIs was highest in the summer and lowest in the winter in both hurdle and steeplechase starts. Good to firm going had the highest incidence of LTIs in both hurdle and steeplechase starts, as well as the highest incidence of TLI in hurdle starts. Good going had the highest incidence of bone injuries in both hurdle and steeplechase starts, as well as the highest incidence of TLI in steeplechase starts. Horses that fell in the race had a higher incidence of LTIs and bone injuries compared to those that did not fall, but the incidence of TLI was higher in horses that did not fall.

## 4 DISCUSSION

This is the first study to report the types and incidences of injuries defined as ‘long‐term’ in jump racing Thoroughbreds at British racecourses. The incidence of LTIs was 5.3 per 1000 starts in hurdle races and 6.1 per 1000 starts in steeplechase races. It is difficult to compare these incidence rates to previous studies, as these tend to report tendon and ligament injuries separately from other injuries or report on all veterinary events including those that are non‐musculoskeletal^15^, ^29^ or include fatal cases.^2^, ^27^, ^28^ That said, the incidence reported here is fairly similar to the incidence of fatality recently reported by Allen et al. (2024) for jump racehorses in GB of 5.9 and 4.5 per 1000 steeplechase and hurdle starts, respectively.^31^

Tendon and ligament injuries made up the majority of LTIs. It is well‐established that these types of injuries tend to require lengthy rehabilitation periods^20^ and often result in premature retirement from racing^10^, ^14^ due to a high risk of re‐injury^21^, ^22^, ^23^ or because the prognosis for returning to racing is poor.^14^, ^24^ However, the ability to treat tendon and ligament strains largely conservatively without the significant financial costs of surgery common in fracture cases, as well as a greater variability in injury severity, may explain the higher proportion of non‐fatal TLIs. In contrast, fractures make up a higher proportion of fatal injuries in comparison to TLIs in jump racing.^31^

Tendon and ligament injuries were most commonly reported as ‘severe (breakdown/ rupture)’ in hurdle starts (45.6%), but ‘moderate’ strains were most commonly reported in steeplechase starts (41.3%). Moderate strains have been the most common injury type reported previously in jump racing,^6^ NHF racing,^29^ and flat racing^15^ in GB. However, ‘severe’ TLIs may have been identified more frequently in hurdle starts as hurdle races tend to be run at faster speeds than steeplechase races due to smaller obstacles and shorter distances. Furthermore, a greater number of ex‐flat horses tend to compete in hurdle races, and horses with a higher proportion of their career spent racing on the flat have been associated with an increased likelihood of SDF tendinopathy in hurdle starts previously.^27^ A greater number of flat starts has also been reported as a risk factor for fatality in jump racehorses in Victoria, Australia.^32^ Injury severity may be higher in these horses as they have experienced an increased number of high‐speed loading cycles throughout their flat racing career, allowing damage to accumulate and weaken the tendons and ligaments prior to clinical injury.^33^, ^34^, ^35^ However, Ely et al. (2009) found that ex‐store horses (horses bred specifically for jump racing) were twice as likely as ex‐flat horses to incur a TLI on the racecourse,^36^ which may reflect a ‘healthy horse effect’, where only flat horses whose tendons withstood flat racing transition into jump racing.^27^, ^36^ Alternatively, this may be because ex‐store horses are typically older when starting their career. Horses participating in flat races can run as two‐year‐olds, whereas jump races, including NHF races, are restricted to horses aged 3 years or older. Horses that began their careers in NHF races have been found to be at a higher risk of SDF tendinopathy in hurdle races.^27^ Therefore, more severe injuries may be seen in hurdle racing due to the increased numbers of younger and less experienced horses beginning their racing careers, whose tendons and ligaments are not adapted to the increased loads experienced in racing compared to training.

That said, injury severity classification may not be highly accurate, as in many cases diagnosis was based on clinical examination alone, without ultrasound imaging, and so may have varied between examining veterinary surgeons.^6^ Similar to previous studies,^2^, ^6^, ^25^, ^27^, ^28^ injuries to the SDFT were found to be the most common type of TLI in jump racehorses, and LTI in this study. Racing places the SDFT under strains near its functional limit,^37^ which can lead to cumulative microdamage, predisposing horses to clinical injury.^38^, ^39^ Jump racehorses tend to be older than flat racehorses, compete over longer distances over more seasons, increasing their risk of injury,^2^, ^36^, ^40^, ^41^, ^42^ and their tendons are also likely to be placed under higher strains when landing over fences.

The incidence reported here for bone injuries included cases of possible fractures (40.8%). Racecourse veterinary identification of fracture presence has previously been found to be at least 93% accurate based on subsequent post‐mortem examination,^43^ and therefore it is likely that many of these ‘possible fracture’ cases were indeed fractures. Digital and wireless radiography mean that diagnostic imaging is often available at the racecourse, providing an advantage in determining precise fracture locations and configurations, planning necessary support and obtaining secondary opinions on management and prognosis.^44^ However, whilst the availability of diagnostic imaging at racecourses may have increased, in some cases it is in the best interests of the horse for it to be transferred to a hospital as soon as possible, or home, where their usual veterinary surgeon can manage the horse and therefore only presumptive diagnoses based on clinical examination can be recorded. Veterinary officers are now following up these cases after further investigation has taken place, by contacting trainers, helping to confirm the subsequent diagnosis and allowing details to be updated in the BHA database.

Previous studies have identified fractures of the MC3/MT3 to be the most frequently affected distal limb structure in jump racing,^6^, ^36^ NHF racing,^29^ and flat racing^15^ in GB. This study separated condylar fractures of the MC3/MT3 from other fracture types to the same bone (*n* = 5), but even if these categories were combined, ACB fracture was still the most common distal limb bone injury in both steeplechase and hurdle starts. This is likely because a substantial proportion of MC3/MT3 fractures are fatal and therefore not captured under the long‐term injury definition; MC3 lateral condylar fractures have previously been identified to be the most common fatal distal limb fracture in National Hunt racing.^16^ Though ACB fractures are considered to be relatively uncommon, they occur most frequently in racehorses and event horses when jumping over fences,^45^, ^46^ hence their commonality in this study. These fractures are thought to occur via either a bow string effect on the bone exerted through the pull of the ulnaris lateralis, the flexor carpi ulnaris and the deep and SDFT muscles when the horse lands on a partially flexed limb or during hypertension,^47^, ^48^ or alternatively as a result of being trapped in a ‘nutcracker’ between the MC3 and the radius,^49^ for example after a fall. For 74.3% (29/39) of ACB fracture cases in this study, the horse fell, unseated the jockey or slipped up. Pelvic fractures were the most common proximal limb fracture in both steeplechase and hurdle starts, comparable to previous studies in flat and NHF racing populations in GB.^15^, ^29^ The incidence of pelvic fracture was higher in steeplechase than hurdle races, a similar relationship to that identified by Reardon (2013) in jump racehorses in GB.^6^

The highest incidence of LTIs across the study period was reported in the 2018/19 racing year, whereas the lowest incidences of LTIs across the study period were reported in 2019/2020 and 2020/21. These two racing years were affected by the COVID‐19 pandemic ‘lockdown’ restrictions, when jump racing in GB was suspended from 18 March 2020 and did not resume until 1 July 2020.^50^ This may have resulted in horses having fewer runs, an extended summer break, or older horses, who have been found to be at greater risk of MSI,^2^, ^15^, ^28^, ^36^, ^40^, ^41^, ^42^, ^51^ being retired voluntarily. Horses may have been kept in work as it was unclear as to when racing would return, without the extra stresses associated with racing and therefore allowing for more time to adapt to loads, better preparing them for the following racing season. However, a detailed investigation into how horses' racing and training schedules were affected during that time would be necessary to fully understand the impacts of the COVID‐19 pandemic.

The incidence of LTIs, bone injuries and TLIs was highest in the summer and lowest in the winter in both hurdle and steeplechase starts, comparable to findings in NHF races for bone injuries and TLIs^29^ and with studies assessing risk factors for SDF tendinopathy in hurdle and steeplechase racing^27^, ^28^ and hindlimb fracture in steeplechase starts^6^ in GB. These studies found this to be the case when accounting for racecourse going,^27^, ^28^, ^31^ therefore, seasonal differences have been attributed to higher summer temperatures, possibly contributing to fatigue, other ground surface factors potentially caused by artificial watering^27^, ^31^ and differences in the experience and ability of horses racing in the summer compared with the core jump season (October to April). The finding that the incidence of LTIs increased with firmer going in both steeplechase and hurdle races is consistent with previous studies assessing risk factors for MSIs and fatalities in GB,^2^, ^6^, ^31^, ^52^ SDF tendinopathy in steeplechase and hurdle starts,^27^, ^28^ and fracture risk.^1^, ^2^, ^6^, ^52^, ^53^, ^54^ However, there was little difference between the incidence of bone injuries across the different goings in the current study. The incidence of LTIs, bone injuries and TLIs in females was higher than in males in both hurdle and steeplechase starts. Females have been found to have significantly lower odds of fatality compared to males in jump racing,^31^ possibly as connections of female horses may be more willing to salvage a mare for breeding purposes.^51^ Whereas, geldings offer no breeding potential and if they are unsuitable for an alternative career or their previous racing performance was poor, they may be more likely to be electively euthanised. The incidence of LTI and TLI in steeplechase starts increased with increasing age, in agreement with previous findings in National Hunt horses in training in GB.^36^, ^40^, ^42^ Age has also been identified as a risk factor for SDF tendinopathy in steeplechase starts in the UK.^28^ Fractures have not consistently been associated with increasing age in numerous studies,^36^, ^55^, ^56^, ^57^, ^58^, ^59^, ^60^ because of different injury pathways (fractures occurring in unadapted and adapted bone). Similarly, there was no clear relationship with age and the incidence of bone injuries in this study. The incidence of LTIs and bone injuries was higher in horses that fell in the race, but the incidence of TLI was higher in horses that did not fall. Ely et al. (2009) also reported a higher percentage of bone injuries associated with falls compared to TLIs in jump racehorses in GB.^36^ Numerous studies have investigated risk factors for falling in jump racing,^61^, ^62^, ^63^, ^64^, ^65^, ^66^, ^67^, ^68^ but the role of faller status as a risk factor for injury has been less frequently examined. This is likely due to the difficult nature of determining whether the fall was the result of, or resulted in, injury.^31^ A recent study on fatality risk in jump racing in GB highlighted a strong significant association between faller status and the odds of fatality, likely because fatalities are often associated with falling.^31^ The strength of this association and the incidence rates reported here suggest that this is an important risk factor that should be considered in future epidemiological risk factor studies relating to jump racehorses. Furthermore, previous work identifying risk factors for falling may also help to reduce fatality and injury.^31^

Further multivariable analyses will enable quantification of the associations between the stratified variables reported here and specific outcomes, when adjusted for the effects of potential confounders, but were beyond the scope of this study. It also remains unclear whether a 90‐day break from racing is the most appropriate definition for an LTI, and a consensus across jurisdictions is needed to facilitate meaningful comparisons. The categorisation of LTI was based on race‐day reports and recording systems developed by the BHA. Given the limited use of diagnostic imaging whilst the horse is at the racecourse, some reported injuries were based on the signs identified during a clinical examination rather than on a definitive diagnosis. Horses also may have left the racecourse before an injury was diagnosed, even when it was sustained during racing, and therefore, LTIs may have been under‐reported or misclassified based on the presenting signs at the time of the event. Consequently, it is likely that incidence rates reported here are underestimates of the true incidence of race‐related LTIs. Additionally, horses that were electively euthanised, having not met the BEVA guidelines for immediate humane destruction,^69^ would have been classified as fatalities and not included under the LTI definition, even though they may have had similar injuries to the cases in this study. This may have contributed to certain injury types being under‐reported. However, these cases cannot be classified as LTIs as they do not have the potential to return to racing. Furthermore, it is not known if the LTI itself was the direct cause of the horse's extended break from racing, particularly in cases where the diagnosis was presumptive. The horse may have suffered an injury or setback in training, or the injury may have coincided with the horse's planned seasonal break, resulting in a longer time away from the racecourse. This highlights the importance of Veterinary Officers' mandatory pre‐race examination of horses returning after race‐related LTIs and/or an extended break, and determining and recording the reason for the horse's extended break from racing.

This study has provided up‐to‐date estimates of the incidence of race‐related LTIs in jump racing Thoroughbreds in GB. The incidence of LTIs was higher in steeplechase starts than hurdle starts, and TLIs were most commonly reported, followed by bone injuries. The findings from this study will now inform further multivariable statistical analysis to determine risk factors for LTIs. These results also provide a baseline against which to measure the effect of potential interventions aimed at reducing injury occurrence in jump racing in GB.

## FUNDING INFORMATION

The Royal Veterinary College's Mellon Fund for Equine Research and The Racing Foundation through the Horse Welfare Board.

## CONFLICT OF INTEREST STATEMENT

The authors declare no conflicting interests.

## AUTHOR CONTRIBUTIONS

**Sophia McDonald:** Writing – original draft; writing – review and editing; formal analysis. **Kristien L. P. Verheyen:** Conceptualization; methodology; writing – review and editing; supervision; funding acquisition. **Yu‐Mei Chang:** Conceptualization; methodology; writing – review and editing; supervision. **Sarah E. Allen:** Conceptualization; methodology; writing – review and editing; supervision; funding acquisition.

## DATA INTEGRITY STATEMENT

Sophia McDonald and Sarah E. Allen had full access to all the data in the study and take responsibility for the integrity of the data and the accuracy of data analysis.

## ETHICAL ANIMAL RESEARCH

Ethical approval was obtained from the Royal Veterinary College's Social Science Research Ethical Review Board (URN SR2024‐02611003).

## INFORMED CONSENT

Representatives of the British Horseracing Authority gave consent for the use of their data in this research.

## Supporting information


**Table S1:** Incidence of long‐term injury (LTI), bone injury and tendon and ligament injury (TLI) events experienced by steeplechase and hurdle racing Thoroughbreds in Great Britain as reported by race‐day veterinary surgeons between 1 May 2018 and 30 April 2024, stratified by sex, age, going, season and faller status. Associated 95% confidence intervals (CIs) were calculated for each incidence rate.

## Data Availability

The data that support the findings of this study are available from the British Horseracing Authority. Restrictions apply to the availability of these data, which were used under licence for this study.
