# Supplementary material for: Descriptive epidemiology of long‐term injuries in jump racing Thoroughbreds in Great Britain
Source: Equine Vet J. 2025 Nov 24;58(5):1228–39. doi: 10.1111/evj.70129 (PMC13447888; doi:10.1111/evj.70129)
Supplement: Supplementary file 1 [file EVJ-58-1228-s001.pdf]

Equine Veterinary Journal BEVA

Table S1: Incidence of long-term injury (LTI), bone injury and tendon and ligament injury (TLI) events experienced by steeplechase and hurdle racing Thoroughbreds in Great Britain as reported by race-day veterinary surgeons between 1 May 2018 and 30 April 2024, stratified by sex, age, going, season and faller status. Associated 95% confidence intervals (CIs) were calculated for each incidence rate.

**Variable** Category	Starts, n (%)		Incidence per 1000 starts (95% CI)					
			Hurdle			Steeplechase		
	Hurdle	Steeplechase	LTI	Bone	TLI	LTI	Bone	TLI
**Age (years)**								
3-5	41,339 (38.8)	2,959 (5.0)	3.8 (3.2-4.4)	1.0 (0.7-1.3)	2.6 (2.1-3.1)	4.1 (2.1-7.1)	2.0 (0.7-4.4)	2.0 (0.7-4.4)
6-8	53,034 (49.8)	35,048 (58.6)	5.8 (5.2-6.5)	1.3 (1.0-1.6)	4.2 (3.7-4.8)	5.5 (4.8-6.3)	1.4 (1.1-1.9)	3.8 (3.2-4.5)
9-11	11,079 (10.4)	19,128 (32.0)	8.4 (6.5-9.9)	1.2 (0.6-2.0)	6.6 (5.2-8.3)	7.0 (5.8-8.2)	1.3 (0.8-1.9)	5.2 (4.2-6.3)
≥12	971 (0.9)	2,632 (4.4)	5.1 (1.7-11.9)	1.0 (0.03-5.7)	4.1 (1.1-10.5)	8.4 (5.2-12.6)	1.5 (0.4-3.9)	6.8 (4.1-10.8)
**Sex**								
Male	80,059 (75.2)	53,783 (90.0)	5.1 (4.6-5.6)	1.1 (0.9-1.4)	3.7 (3.3-4.1)	6.0 (5.3-6.7)	1.4 (1.1-1.7)	4.2 (3.7-4.8)
Female	26,364 (24.8)	5,984 (10.0)	5.7 (4.9-6.7)	1.3 (0.9-1.8)	4.2 (3.5-5.1)	6.5 (4.6-8.9)	1.5 (0.7-2.9)	4.8 (3.2-7.0)
**Season**								
Spring	29,223 (27.5)	17,504 (29.3)	5.7 (4.9-6.7)	1.2 (0.9-1.7)	4.4 (3.7-5.2)	7.0 (5.8-8.4)	1.9 (1.3-2.6)	4.7 (3.7-5.8)
Summer	14,215 (13.4)	7,514 (12.6)	8.0 (6.6-9.6)	1.7 (1.1-2.5)	6.1 (4.9-7.5)	9.7 (7.6-12.2)	2.1 (1.2-3.5)	7.1 (5.3-9.2)
Autumn	29,461 (27.7)	15,674 (26.2)	5.6 (4.8-6.6)	1.1 (0.7-1.5)	4.3 (3.6-5.1)	6.6 (5.4-8.0)	1.5 (1.0-2.3)	4.6 (3.6-5.8)
Winter	33,524 (31.5)	19,075 (31.9)	3.3 (2.7-4.0)	0.9 (0.6-1.3)	1.9 (1.5-2.5)	3.1 (2.4-4.0)	0.6 (0.3-1.0)	2.5 (1.8-3.3)
**Going**								
Heavy	9,243 (8.7)	5,528 (9.3)	2.5 (1.6-3.7)	1.0 (0.4-1.8)	1.2 (0.6-2.1)	2.5 (1.4-4.2)	0.9 (0.3-2.1)	1.4 (0.6-2.8)
Soft	25,446 (23.9)	15,284 (25.6)	2.8 (2.1-3.5)	0.7 (0.4-1.1)	1.7 (1.3-2.3)	4.3 (3.3-5.4)	1.3 (0.8-2.0)	2.8 (2.0-3.8)
Good to soft	26,423 (24.8)	14,348 (24.0)	4.1 (3.4-4.9)	1.2 (0.8-1.7)	2.8 (2.2-3.5)	5.5 (4.4-6.9)	1.0 (0.5-1.6)	3.8 (2.8-4.9)
Good	42,957 (40.4)	23,441 (39.2)	7.7 (6.9-8.6)	1.4 (1.1-1.8)	6.0 (5.3-6.7)	8.2 (7.1-9.4)	1.8 (1.3-2.5)	6.1 (5.1-7.1)
Good to firm	2,332 (2.2)	1,159 (1.9)	11.6 (7.6-16.8)	1.3 (0.3-3.8)	9.4 (5.9-14.2)	8.6 (4.1-15.8)	1.7 (0.2-6.2)	6.0 (2.4-12.4)
Firm	22 (<0.1)	7 (<0.1)	0.0 (0.0-15.4)	0.0 (0.0-154.4)	0 (0.0-154.4)	0.0 (0.0-409.6)	0.0 (0.0-409.6)	0.0 (0.0-409.6)
**Horse fell in race**								
Yes	1,860 (1.7)	2,001 (3.3)	24.7 (18.2-32.9)	18.8 (13.1-26.1)	1.6 (0.3-4.7)	20.5 (14.7-27.7)	17.5 (12.2-24.2)	2.5 (0.8-5.8)
No	104,563 (98.3)	54,766 (97.0)	4.9 (4.5-5.3)	0.8 (0.7-1.0)	3.9 (3.5-4.3)	5.8 (5.2-6.5)	0.9 (0.6-1.2)	4.5 (4.0-5.1)
